# Respiratory symptoms among infants at risk for asthma: association with surfactant protein A haplotypes

**DOI:** 10.1186/1471-2350-8-15

**Published:** 2007-04-02

**Authors:** Melinda M Pettigrew, Janneane F Gent, Yong Zhu, Elizabeth W Triche, Kathleen D Belanger, Theodore R Holford, Michael B Bracken, Brian P Leaderer

**Affiliations:** 1 Yale Center for Perinatal, Pediatric and Environmental Epidemiology, Department of Epidemiology and Public Health, Yale University School of Medicine, New Haven, CT, USA

## Abstract

**Background:**

We examined the association between single nucleotide polymorphisms (SNPs) in loci encoding surfactant protein A (SFTPA) and risk of wheeze and persistent cough during the first year of life among a cohort of infants at risk for developing asthma.

**Methods:**

Between September 1996 and December 1998, mothers of newborn infants were invited to participate if they had an older child with clinician-diagnosed asthma. Each mother was given a standardized questionnaire within 4 months of her infant's birth. Infant respiratory symptoms were collected during quarterly telephone interviews at 6, 9 and 12 months of age. Due to the association of *SFTPA *polymorphisms and race/ethnicity, analyses were restricted to 221 white infants for whom whole blood and respiratory data were available. Ordered logistic regression models were used to examine the association between respiratory symptom frequency and *SFTPA *haplotypes.

**Results:**

The 6A allele haplotype of *SFTPA1*, with an estimated frequency of 6% among our study infants, was associated with an increased risk of persistent cough (OR 3.69, 95% CI 1.71, 7.98) and wheeze (OR 4.72, 95% CI 2.20, 10.11). The 6A/1A haplotype of *SFTPA*, found among approximately 5% of the infants, was associated with an increased risk of persistent cough (OR 3.20, 95% CI 1.39, 7.36) and wheeze (OR 3.25, 95% CI 1.43, 7.37).

**Conclusion:**

Polymorphisms within *SFTPA *loci may be associated with wheeze and persistent cough in white infants at risk for asthma. These associations require replication and exploration in other ethnic/racial groups.

## Background

Wheeze and persistent cough in infants are serious respiratory symptoms that can be triggered by respiratory infections and/or a variety of environmental exposures [1-3]. Surfactant protein A (SFTPA) is an abundant, multifunctional protein that is secreted by airway epithelial cells and functions as part of the innate immune response. SFTPA may be critical in protecting the lungs from infectious agents and environmental exposures early in life before the acquisition of specific immunity. SFTPA neutralizes respiratory viruses such as influenza and respiratory syncytial virus (RSV) [4,5]. SFTPA also enhances the uptake of bacteria and viruses by phagocytes [6-8]. In addition to its role in protecting the lungs from microorganisms, SFTPA has other important immunomodulatory functions including binding aeroallergens [9].

SFTPA's role in protection of the lungs has led to exploration of potential links between SFTPA and diseases of the respiratory tract in infants and young children. Polymorphisms within *SFTPA1 *and *SFTPA2*, two functional, highly homologous *SFTPA *genes [10-12] have been linked to respiratory distress syndrome in infants [13-15], severe RSV bronchiolitis [16], and otitis media [17,18]. In the present study, we used a candidate gene approach to investigate whether polymorphisms within the *SFTPA1 *and *SFTPA2 *genes were associated with wheeze and persistent cough during the first year of life among a prospective birth cohort at risk for developing asthma.

## Methods

### Cohort

Between September 1996 and December 1998, we invited women delivering babies in six hospitals in Connecticut and Massachusetts to participate in a longitudinal study of asthma development if they had at least one other child at home under 12 years of age with physician-diagnosed asthma. Infants enrolled in the cohort were considered at risk for asthma due to their asthmatic siblings. We provide a full description of the methods elsewhere [2,19].

Of the 1,002 infants originally enrolled, respiratory symptom data were available for the first year of life for 889. Between the third and fifth year of participation, we made a second visit to the home to collect a blood sample from our cohort subjects. The current analyses are based on a convenience sample of 355 for whom whole blood and complete respiratory symptom data were available. Nucleotide changes at amino acid (aa) 19, aa 62, and aa 133 in *SFTPA1*, and aa 223 in *SFTPA2 *were significantly associated with ethnicity. To prevent identification of invalid associations due to population stratification, we restricted our haplotype analyses to the ethnic group with the largest number of subjects (221 white infants). The Yale Human Investigations Committee and institutional review boards at each participating hospital reviewed and approved the study.

### Data collection

A research assistant visited the home within 4 months of the infant's birth in order to describe the study to the infant's mother, obtain written informed consent, and administer a standardized questionnaire. We collected household demographic data including maternal race and ethnicity, education and number of children; detailed information regarding infant care (e.g. breastfeeding and daycare use); and maternal health status (e.g. self-reported history of allergies or physician-diagnosed asthma). Infant respiratory symptoms were collected during quarterly telephone interviews at 6, 9 and 12 months of age at which time each mother reported on her infant's respiratory symptoms (number of symptom-days per month of wheeze or persistent cough) and doctor or clinic visits (month and year of visit, reason for visit, and diagnosis). Around the time of the infant's first birthday, the mother completed an additional phone questionnaire covering her infant's health status during the previous year.

### Genotyping of SFTPA genes

DNA was extracted from whole blood using the QIAamp DNA blood minikit (Qiagen) according to the manufacturer's instructions. We used sequence specific primer-PCR methodology to genotype the SNPs at aa 19, aa 50, aa 62, aa 133, and aa 219 in *SFTPA1 *and aa 9, aa 91, aa 223 in *SFTPA2 *[20]. We used PCR based cRFLP as described by DiAngelo et al. [21] to detect polymorphisms in *SFTPA2 *at aa 140.

### Data analysis

**Figure 1:**
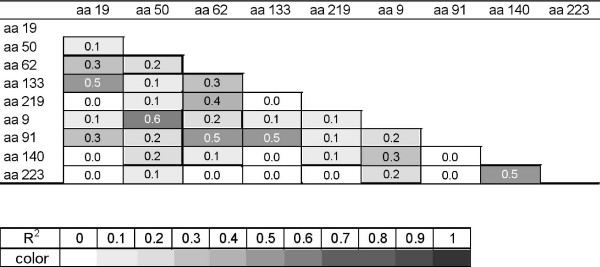
Pairwise linkage disequilibrium measure (r^2^) for surfactant protein A SNPs (5 *SFTPA1 *SNPs: aa 19, aa 50, aa 62, aa 133, aa 219; 4 *SFTPA2 *SNPs: aa 9, aa 91 aa 140, aa 223) from 221 white infants at risk for developing asthma. Gray scale represents strength of association from white (r^2 ^= 0) to black (r^2 ^= 1). (CT and MA, 1998 – 2000)

Outcomes of interest were respiratory symptom frequency in the first year of life categorized as none or as 1 to 7, 8 to 14, 15 to 21, 22 to 28, or more than 28 days of persistent cough or wheeze. Between 1 and 6 months of symptom information were missing for 18 infants. Data from these infants were included in analyses based on their symptom rates. All other infants had 12 months of complete symptom data. Unadjusted associations between respiratory symptoms and selected study characteristics, SNPs, and individual *SFTPA1 *or *SFTPA2 *alleles were evaluated with tests for linear trend (Cochran-Armitage or Somers' D statistic). For statistical tests involving SNPs, frequencies for the minor homozygous allele and heterozygous alleles were combined and compared to the dominant homozygous group. Observed SNP frequencies were tested for Hardy-Weinberg equilibrium by χ^2 ^analyses. We examined pairwise linkage disequilibrium with r^2 ^measures (Fig. 1) using PROC ALLELE of the SAS/Genetics module of SAS version 9.1 [22]. Allele haplotypes for *SFTPA1 *and for *SFTPA2 *were assigned and examined for linkage disequilibrium using PROC HAPLOTYPE [22]. We examined the association between haplotype and respiratory symptom frequency (as categorical variables) using ordered logistic regression. Separate analyses were conducted for each symptom and each haplotype compared to all other haplotypes. We estimated the effect of uncertainty in haplotype assignment using a regression calibration technique [18,23] and the *SFTPA1 *and *SFTPA2 *haplotype probabilities obtained from PROC HAPLOTYPE. As described previously [18], this technique involved first creating 100 separate haplotype data sets by randomly assigning subject haplotypes based on each subject's haplotype probabilities. Next, 100 separate ordered logistic regression models with coefficient and variance estimates (β_i _and σ_i _^2 ^for the i^th ^model) were fit for each haplotype of interest. The estimate of the association between the respiratory symptom and the particular haplotype of interest ( β^
 MathType@MTEF@5@5@+=feaafiart1ev1aaatCvAUfKttLearuWrP9MDH5MBPbIqV92AaeXatLxBI9gBaebbnrfifHhDYfgasaacH8akY=wiFfYdH8Gipec8Eeeu0xXdbba9frFj0=OqFfea0dXdd9vqai=hGuQ8kuc9pgc9s8qqaq=dirpe0xb9q8qiLsFr0=vr0=vr0dc8meaabaqaciaacaGaaeqabaqabeGadaaakeaaiiGacuWFYoGygaqcaaaa@2E64@) was calculated as the mean of the 100 regression models. The variability of the estimate was calculated as

var (β^
 MathType@MTEF@5@5@+=feaafiart1ev1aaatCvAUfKttLearuWrP9MDH5MBPbIqV92AaeXatLxBI9gBaebbnrfifHhDYfgasaacH8akY=wiFfYdH8Gipec8Eeeu0xXdbba9frFj0=OqFfea0dXdd9vqai=hGuQ8kuc9pgc9s8qqaq=dirpe0xb9q8qiLsFr0=vr0=vr0dc8meaabaqaciaacaGaaeqabaqabeGadaaakeaaiiGacuWFYoGygaqcaaaa@2E64@) = mean (σi2
 MathType@MTEF@5@5@+=feaafiart1ev1aaatCvAUfKttLearuWrP9MDH5MBPbIqV92AaeXatLxBI9gBaebbnrfifHhDYfgasaacH8akY=wiFfYdH8Gipec8Eeeu0xXdbba9frFj0=OqFfea0dXdd9vqai=hGuQ8kuc9pgc9s8qqaq=dirpe0xb9q8qiLsFr0=vr0=vr0dc8meaabaqaciaacaGaaeqabaqabeGadaaakeaaiiGacqWFdpWCdaqhaaWcbaGaemyAaKgabaGaeGOmaidaaaaa@30F0@) + var (b_i_s)

where mean (σi2
 MathType@MTEF@5@5@+=feaafiart1ev1aaatCvAUfKttLearuWrP9MDH5MBPbIqV92AaeXatLxBI9gBaebbnrfifHhDYfgasaacH8akY=wiFfYdH8Gipec8Eeeu0xXdbba9frFj0=OqFfea0dXdd9vqai=hGuQ8kuc9pgc9s8qqaq=dirpe0xb9q8qiLsFr0=vr0=vr0dc8meaabaqaciaacaGaaeqabaqabeGadaaakeaaiiGacqWFdpWCdaqhaaWcbaGaemyAaKgabaGaeGOmaidaaaaa@30F0@) is defined as the mean of the 100 logistic regression variances and var (b_i_s) is defined as the variance of the 100 logistic regression b coefficients.

## Results

Close to half of the 221 infants in our study experienced persistent cough (51%) or wheeze (46%) during their first year of life (Table 1). Thirty-two percent of the infants experienced from 1 to 28 days of persistent cough, 19% experienced more than 28 days of persistent cough during their first year; 36% experienced 1 to 28 days of wheeze and 10% experienced more than 28 days of wheeze during their first year of life. Male infants and infants of asthmatic mothers were more likely to experience persistent cough and wheeze during their first year of life (Table 1). We did not find significant associations with either persistent cough or wheeze and maternal allergies, age daycare attendance began, number of months of breastfeeding, or exposure to environmental tobacco smoke (ETS).

**Table 1:** Unadjusted associations between personal characteristics and respiratory symptoms during the first year of life for 221 white infants at risk for developing asthma. (CT and MA, 1998 – 2000)

		**Persistent cough (days/year) (%)**							**Wheeze (days/year) (%)**						
**Characteristic**	**n (%)**	**0**	**1 – 7**	**8 – 14**	**15 – 21**	**22 – 28**	**> 28**	**p-value**^a^	**0**	**1 – 7**	**8 – 14**	**15 – 21**	**22 – 28**	**> 28**	**p-value**^a^

**White infants N (%)**	**221**	109 (49.3)	30 (13.6)	19 (8.6)	12 (5.4)	10 (4.5)	41 (18.6)		119 (54.3)	49 (22.4)	17 (7.8)	7 (3.2)	6 (2.7)	21 (9.6)	
**Gender**															
male	115 (52.0)	42.6	14.8	11.3	7.0	7.0	17.4	0.12	44.2	27.4	8.0	3.5	4.4	12.4	**0.005**
female	106 (48.0)	56.6	14.3	5.7	3.8	1.9	19.8		58.0	17.0	7.6	2.8	0.9	6.6	
**Maternal allergies**															
No	81 (36.7)	44.4	17.3	8.6	3.7	2.5	23.5	0.20	52.5	21.2	10.0	3.8	2.5	10.0	0.35
Yes	140 (63.4)	52.1	11.4	8.6	6.4	5.7	15.7		55.4	23.0	6.5	2.9	2.9	9.4	
**Maternal asthma**															
No	153 (69.2)	52.3	15.0	9.2	5.2	2.0	16.3	**0.02**	57.2	23.7	7.9	2.6	0.7	7.9	**0.01**
Yes	68 (30.7)	42.6	10.3	7.4	5.9	10.3	23.5		47.8	19.4	7.5	4.5	7.5	13.4	
**Daycare (starting age in months)**															
1 – 5	43 (19.6)	48.8	14.0	9.3	2.3	7.0	18.6	0.26	55.8	14.0	11.6	2.3	4.6	11.6	0.45
6 – 12	21 (9.6)	57.1	9.5	19.0	4.8	0.0	9.5		55.0	30.0	10.0	0.0	0.0	0.5	
None in first year	155 (70.8)	49.0	14.2	6.4	6.4	4.5	19.4		53.9	24.0	6.5	3.9	2.6	9.1	
**Breast feeding (duration in months)**															
None	49 (22.3)	53.1	14.3	6.1	4.1	0.0	22.4	0.25	49.0	18.4	10.2	2.0	6.1	14.3	0.15
1 – 5	57 (25.9)	49.1	19.3	3.5	7.0	5.3	15.8		57.9	19.3	3.5	3.5	1.8	14.0	
6 – 12	114 (51.8)	47.4	10.5	12.3	5.3	6.1	18.4		54.5	25.9	8.9	3.6	1.8	5.4	
**ETS**															
No	197 (89.6)	48.7	12.2	9.6	5.6	4.6	19.3	0.19	55.4	20.5	8.7	3.1	3.1	9.2	0.38
Yes	23 (10.4)	52.2	26.1	0.0	4.4	4.4	13.0		43.5	39.1	0.0	4.4	0.0	13.0	

^a^p-values from test for trend: Cochran-Armitage exact test (1-sided) for gender, maternal allergies, maternal asthma, Environmental Tobacco Smoke (ETS); Somer's D statistic (1-sided) for age when starting daycare and duration of breastfeeding. Significant p-values (p < 0.05) in bold-face type.

Table 2 contains unadjusted associations between each of the nine candidate SNPs and persistent cough or wheeze. Individual SNPs were in Hardy-Weinberg equilibrium (p > 0.05 for the χ^2 ^test). Having a C nucleotide at aa 19 in *SFTPA1 *was associated with greater frequency of wheeze. The G nucleotide at aa 133 was associated with both wheeze and persistent cough during the first year of life. We did not find any significant associations between each of the nine SNPs and personal characteristics listed in Table 1.

**Table 2:** Unadjusted associations between SNPs from surfactant protein A alleles (*SFTPA1*,*SFTPA2*) and respiratory symptoms^a ^during the first year of life for white infants at risk for developing asthma. (CT and MA, 1998 – 2000)

		**Persistent cough (days/year) (%)**							**Wheeze (days/year) (%)**						
**Characteristic**	**n (%)**	**0**	**1 – 7**	**8 – 14**	**15 – 21**	**22 – 28**	**> 28**	**p-value**^b^	**0**	**1 – 7**	**8 – 14**	**15 – 21**	**22 – 28**	**> 28**	**p-value**^b^

**White infants N (%)**	**221**	109 (49.3)	30 (13.6)	19 (8.6)	12 (5.4)	10 (4.5)	41 (18.6)		119 (54.3)	49 (22.4)	17 (7.8)	7 (3.2)	6 (2.7)	21 (9.6)	
***SFTPA1 ***^c^															
**aa 19 (rs1059047)**															
CC	1 (0.4)														
CT	42 (19.0)	46.5	7.0	11.6	7.0	2.3	25.6	0.15	44.2	18.6	11.6	4.6	7.0	14.0	**0.02**
TT	178 (80.5)	49.7	14.9	8.0	5.1	5.1	17.1		56.6	23.1	6.9	2.9	1.7	8.7	
**aa 50 (rs1136450)**															
CC	48 (21.4)														
CG	103 (46.0)	51.7	10.9	8.2	6.1	3.4	19.7	0.50	53.4	22.6	8.9	2.7	3.4	8.9	0.51
GG	73 (32.6)	45.2	17.8	9.6	4.1	6.8	16.4		55.6	22.2	5.6	4.2	1.4	11.1	
**aa 62 (rs1136451)**															
GG	5 (2.2)														
AG	61 (27.5)	45.3	7.8	14.1	6.2	3.1	23.4	0.11	46.9	21.9	10.9	3.1	6.2	10.9	0.07
AA	156 (70.3)	51.3	15.6	6.5	4.6	5.2	16.9		57.9	21.7	6.6	3.3	1.3	9.2	
**aa 133 (rs1059057)**															
GG	1 (0.4)														
AG	38 (16.9)	36.8	7.9	13.2	7.9	2.6	31.6	**0.02**	39.5	18.4	10.5	5.3	10.5	15.8	**0.003**
AA	186 (82.7)	51.9	14.8	7.6	4.9	4.9	15.8		57.5	23.2	7.2	2.8	1.1	8.3	
**aa 219 (rs4253527)**															
TT	1 (0.4)														
CT	34 (15.1)	52.9	11.8	20.6	2.9	0.0	11.8	0.12	55.9	29.4	11.8	0.0	0.0	2.9	0.06
CC	190 (84.4)	48.7	13.9	6.4	5.9	5.4	19.8		54.0	21.1	7.0	3.9	3.2	10.8	
***SFTPA2 ***^c^															
**aa 9 (rs1059046)**															
CC	42 (18.7)														
AC	109 (48.4)	52.4	11.6	9.5	4.1	3.4	19.0	0.26	55.1	21.1	8.8	2.0	3.4	9.5	0.50
AA	74 (32.9)	43.2	17.6	6.8	8.1	6.7	17.6		52.8	25.0	5.6	5.6	1.4	9.7	
**aa 91 (rs17886395)**															
CC	3 (1.4)														
CG	57 (25.7)	50.8	8.5	10.2	5.1	3.4	22.0	0.33	54.2	18.6	6.8	3.4	8.5	8.5	0.23
GG	162 (73.0)	49.1	15.1	8.2	5.7	5.0	17.0		54.8	23.6	8.3	3.2	0.6	9.6	
**aa 140 (rs1965707)**															
TT	13 (5.8)														
CT	87 (38.8)	53.1	15.3	8.2	2.0	2.0	19.4	0.15	53.1	24.5	10.2	3.1	2.0	7.1	0.24
CC	124 (55.4)	45.9	12.3	9.0	8.2	6.6	18.0		55.0	20.8	5.8	3.3	3.3	11.7	
**aa 223 (rs1965708)**															
AA	7 (3.1)														
AC	71 (31.6)	54.0	11.8	9.2	2.7	4.0	18.4	0.27	55.3	23.7	9.2	4.0	1.3	6.6	0.18
CC	147 (65.3)	46.9	14.5	8.3	6.9	4.8	18.6		53.8	21.7	7.0	2.8	3.5	11.2	

^a^N = 221 infants had data for persistent cough and N = 219 for wheeze during the first year of life. ^b^p-values from test for trend: Cochran-Armitage exact test (1-sided). Minor homozygotes and heterozygous alleles were combined and compared to the dominant homozygous group. Significant p-values (p < 0.05) in bold-face type. ^c ^SNPs were in Hardy-Weinberg equilibrium. rs numbers are given in parentheses next to each amino acid.

By convention, *SFTPA1 *and *SFTPA2 *allele haplotypes are denoted by 6A^n ^and 1A^m ^respectively [17,21]. The estimated frequency of each allele haplotype is given in Table 3. The five SNPs in *SFTPA1 *were in linkage disequilibrium as were the four SNPs in *SFTPA2 *(p < 0.0001, χ^2 ^test of allelic associations).

**Table 3:** Unadjusted associations between surfactant protein A (*SFTPA1*,*SFTPA2*) haplotypes and persistent cough or wheeze in the first year of life.^a ^Unadjusted odds ratios (OR) and 95% confidence intervals (CI) are from ordered logistic regression models predicting symptom frequency. ^b ^(CT and MA, 1998 – 2000)

**Allele, haplotype**^c^	**Nucleotide/amino acid**^d^					**Estimated Frequency Distribution^e^% (95% CI)**	**Persistent Cough OR (95% CI)**	**Wheeze OR (95% CI)**
***SFTPA1***	**aa 19**	**aa 50**	**aa 62**	**aa 133**	**aa 219**			
				
**6A**	C/Ala	C/Leu	G	G	C/Arg	6.1% (4.0 – 8.3)	**3.69 (1.71, 7.98)**	**4.72 (2.20, 10.11)**
**6A**^2^	T/Val	G/Val	A	A	C/Arg	54.0% (49.3 – 58.7)	1.18 (0.63, 2.19)	1.13 (0.60, 2.14)
**6A**^3^	T/Val	C/Leu	A	A	C/Arg	25.6% (21.6 – 29.6)	0.81 (0.49, 1.36)	0.83 (0.49, 1.40)
**6A**^4^	T/Val	C/Leu	G	A	T/Trp	6.2% (4.1 – 8.4)	0.94 (0.44, 2.03)	0.70 (0.31, 1.60)
**all others**						8%		
								
***SFTPA2***	**aa 9**	**aa 91**	**aa 140**	**aa 223**				
					
**1A**	C/Thr	C/Pro	C	C/Gln		8.4% (6.0 – 10.8)	1.53 (0.75, 3.14)	1.30 (0.63, 2.71)
**1A**^0^	A/Asn	G/Ala	C	C/Gln		54.2% (49.5 – 58.8)	1.13 (0.60, 2.13)	1.14 (0.59, 2.19)
**1A**^1^	C/Thr	G/Ala	T	A/Lys		13.7% (10.7 – 16.7)	0.82 (0.45, 1.48)	0.87 (0.47, 1.58)
**1A**^2^	C/Thr	G/Ala	C	C/Gln		10.2% (7.6 – 12.7)	0.95 (0.49, 1.84)	0.83 (0.42, 1.64)
**1A**^3^	A/Asn	G/Ala	T	A/Lys		1.8% (0.7 – 2.9)	0.45 (0.08, 2.48)	0.55 (0.10, 3.06)
**1A**^5^	C/Thr	C/Pro	T	C/Gln		4.6% (2.7 – 6.5)	0.43 (0.15, 1.23)	0.66 (0.24, 1.79)
**1A**^6^	C/Thr	G/Ala	T	C/Gln		3.2% (1.7 – 4.7)	1.41 (0.49, 4.05)	1.54 (0.54, 4.39)
**all others**						4%		
								
***SFTPA *Haplotype**								
**6A/1A**						5.4% (3.2 – 7.2)	**3.20 (1.39, 7.36)**	**3.25 (1.43, 7.37)**
**6A**^2^**/1A**^0^						49.0% (44.4 – 53.6)	1.20 (0.66, 2.19)	1.04 (0.57, 1.90)
**6A**^2^**/1A**^2^						1.8% (0.6 – 2.9)	1.43 (0.36, 5.70)	0.26 (0.03, 1.99)
**6A**^2^**/1A**^3^						1.0% (0.2 – 1.9)	0.65 (0.10, 4.39)	0.79 (0.11, 5.47)
**6A**^3^**/1A**^0^						4.7% (2.8 – 6.6)	1.16 (0.49, 2.74)	0.92 (0.37, 2.31)
**6A**^3^**/1A**^1^						11.3% (8.5 – 14.1)	1.12 (0.60, 2.08)	1.08 (0.57, 2.04)
**6A**^3^**/1A**^2^						5.6% (3.6 – 7.6)	0.93 (0.40, 2.16)	0.92 (0.39, 2.17)
**6A**^3^**/1A**^6^						1.7% (0.5 – 2.8)	0.91 (0.21, 4.07)	2.26 (0.55, 9.33)
**6A**^4^**/1A**^5^						2.9% (1.4 – 4.4)	0.28 (0.07, 1.07)	0.30 (0.08, 1.20)
**6A**^4^**/1A**^6^						1.8% (0.6 – 3.0)	1.88 (0.49, 7.16)	1.69 (0.44, 6.39)
**Others**						14.9%		

^a^Analyses included infants with complete data for both *SFTPA *alleles and respiratory symptoms, i.e., for *SFTPA1 *and persistent cough (N = 203) or wheeze (N = 202); *SFTPA2 *and persistent cough (N = 205) or wheeze (N = 204); *SFTPA *and persistent cough (N = 201) or wheeze (N = 200). ^b^Separate ordered logistic regression analyses were performed for each outcome measure and each haplotype with all other haplotypes serving as the reference group in each model. All model results (ORs [95% CI]) include estimates of model variabillity due to ambiguity in allele haplotype assignment (see text). Number of days per year of symptoms were included in models as a 6-category variable, i.e., 0, 1–7, 8–14, 15–21, 22–28, > 28 days. Significant results (p < 0.05) are in bold-face type. ^c ^By convention, *SFTPA1 *allele haplotypes are denoted by 6A^n ^and *SFTPA2 *allele haplotypes by 1A^m ^[17,21]. ^d^aa 62, aa 133 and aa 140 are silent. ^e^Estimated frequency distribution of haplotypes (mean, 95% CI) for white infants.

The most common *SFTPA1 *allele haplotypes among white infants in our study population were 6A^2^, 6A^3^, 6A^4^, and 6A in order of decreasing frequency. All others made up 8% of the *SFTPA1 *alleles. 1A^0 ^was the most common *SFTPA2 *allele followed in order by 1A^1^, 1A^2^, 1A, 1A^5^, 1A^6^, and 1A^3^. All others combined made up 4% of the *SFTPA2 *alleles.

Significant associations were found between specific allele haplotypes and frequency of persistent cough or wheeze during the first year of life (Table 3). The 6A haplotype of *SFTPA1 *was a risk factor for both persistent cough and wheeze: infants with this haplotype (an estimated 6% of this group of white infants) were 3.7 to 4.7 times more likely to experience an additional week of persistent cough or wheeze, respectively, during their first year than infants without this haplotype.

*SFTPA1 *and *SFTPA2 *alleles are known to be in strong linkage disequilibrium [24,25]. This was also true in our population of white infants in a general test of allelic associations (p < 0.0001, χ^2 ^test), although pairwise linkage disequilibrium measures (r^2^) for SNPs within *SFTPA *reveal a spectrum of associations from r^2 ^= 0 to 0.6 (Fig. 1). An examination of *SFTPA1 *and *SFTPA2 *alleles together indicates that infants with the 6A/1A haplotype, an estimated 5.4% of this group, were over 3 times more likely to experience an additional week of persistent cough and/or wheeze during their first year than infants without this haplotype (Table 3).

## Discussion and conclusion

Results from our study suggest that the 6A allele haplotype of *SFTPA1 *and the 6A/1A haplotype of *SFTPA *are associated with increased risk for wheeze and persistent cough among infants at risk for asthma. To our knowledge, this is the first study examining the association of polymorphisms in *SFTPA *with persistent cough and wheeze in infants. Respiratory symptoms may be triggered by bacterial or viral respiratory infections or exposure to environmental contaminants. This is certainly true for children in our birth cohort [2,3,19]. As reported previously [2], among all children in our birth cohort, infants whose mothers reported respiratory illnesses including bronchitis, bronchiolitis, pneumonia, or croup, were 3 to 5 times more likely to experience persistent cough or wheeze in the first year of life than infants who had no respiratory illness in their first year. Among these same infants, respiratory symptoms have also been linked to household exposures such as NO_2 _[3] and mold [2,19].

SFTPA likely plays multiple pleiotropic roles in the pathophysiology of the lung. Evidence from animal and human studies suggests an important role for SFTPA in protecting infants and young children during microbial infections early in life. SFTPA deficient mice are impaired in their ability to clear adenovirus from the lung [24]. SFTPA knockout mice show delayed clearance of *Haemophilus influenzae *[25]. Cell culture assays indicate that SFTPA enhances phagocytosis of *H. influenzae *and *Streptococcus pneumoniae *[26].

Among infants, polymorphisms in *SFTPA *have been associated with severe bronchiolitis [16]. The 6A^2^/1A^3 ^haplotype was associated with increased risk of severe RSV infection (OR 10.4 95% CI 1.3–83.2) and haplotype 6A/1A was protective for severe disease (OR 0.17 95% CI 0.04–0.80). In this study of Finnish infants, cases were hospitalized infants with documented bronchilotis caused by RSV. Controls, matched on sex and age, had no history of respiratory infections requiring hospitalization, but might have had respiratory infections (and symptoms) not requiring hospitalization. Although we identified 6A/1A with increased risk for wheeze and persistent cough, it is interesting that the 6A/1A haplotype is associated with respiratory problems in both populations. Direct comparison between the two studies is difficult because of differences in study design. In our study, "cases" were defined by reported respiratory symptoms. Specific causal agents were not identified, and we did not use hospitalization as a requirement for inclusion. Thus, some of our study subjects could resemble Finnish cases (hospitalized for RSV) or controls (not hospitalized, but possibly suffering from respiratory infection and exhibiting respiratory symptoms).

SFTPA has been shown to bind to aeroallergens including inhalable extracts from the mold *Aspergillus fumigatus *[27] and from the mite *Dermatophagoides pteronyssinus *(*Der p*) [28]. SFTPA has also been postulated to play a role in allergic asthma [29]. A murine model of asthma indicates that SFTPA mRNA and protein levels increase in response to allergen challenge [30]. SFTPA decreases *Der p *induced lymphocyte proliferation and histamine release from the blood of atopic donors [31]. SFTPA has also been implicated in bronchial inflammation of sensitized mice [32].

All infants in our cohort have at least one sibling with asthma and one-third have asthmatic mothers (Table 1). Two-thirds of the infants have mothers with allergies (Table 1). The children in our cohort experienced high rates of wheeze (46%) during their first year of life (Table 1). Among a cohort of 890 healthy infants in Connecticut and Virginia born between 1993–1996, 33% experienced an episode of wheeze during the winter months of their first year of life [34]. The high rate of wheeze in our study population may reflect the special nature of our cohort: all are considered to be at risk for developing asthma. Along these same lines, *SFTPA *may play a role in the pathogenesis of asthma, and infants in our cohort, by virtue of their family histories of asthma, may differ in *SFTPA1 *and *SFTPA2 *hapylotype distributions compared to the general population. Population based estimates of *SFTPA1 *allele haplotype frequencies among white Americans are 56.2% 6A^2^, 24.3% 6A^3^, 9.3% 6A, 7.6% 6A^4 ^and 2.6% other [33]. Allele haplotype frequencies for *SFTPA2 *are 53% 1A^0^, 10.2% 1A, 14.3% 1A^1^, 7.6% 1A^2^, and 14.9% all others [33]. With the notable exception of the *SFTPA1 *allele haplotype 6A, the distribution of *SFTPA1 *and *SFTPA2 *haplotypes in our sample of white infants is similar to that of white Americans in the general population. The general population frequency for 6A of 9.3% is higher than the 95% confidence interval for our estimate of 6.1% (95% CI 4.0–8.3), which may indicate a true difference in frequency for this allele haplotype among our group of infants.

The functional significance of specific changes in nucleotide sequence within *SFTPA *genes has not been well studied. *SFTPA *is a member of the collectin family and recognizes carbohydrates on the surface of pathogens via their carbohydrate recognition domain [35]. Allele 6A is the only common haplotype in our population with an alanine at aa 19 and a G at aa 133. The SNP at aa 133 is silent, however, aa 19 is in the N terminal region of *SFTPA1 *and an alanine in this region could conceivably impact binding to pathogens or aeroallergens. Further experiments are needed to identify whether amino acid changes in this region impact the biological properties of SFTPA. Alternatively, the 6A haplotype may simply be a marker for additional uncharacterized functional polymorphisms in our cohort.

Strengths of this study include the prospective study design and the well characterized demographic, illness, and environmental exposure information for the infant cohort. Mothers in this study were likely capable of accurately reporting respiratory symptoms due to their familiarity with wheeze and persistent cough in the older asthmatic child. Haplotype analyses often use the "most likely" haplotype and seldom include adjustments for uncertainty in haplotype assignment. We used regression calibration techniques that incorporate this uncertainty into estimates of the effect resulting in more conservative estimates of the true associations between *SFTPA *and persistent cough or wheeze during the first year of life.

Our results support the importance of SFTPA in modulating respiratory symptoms in infants. Persistent cough and wheeze may result from a variety of exposures and the multifunctional nature of SFTPA indicates that it protects the lung under a variety of conditions. The 6A/1A haplotype may have a functional role in pathogenic processes, or may be linked to unmeasured markers that are causal. Future studies should replicate these observations and examine polymorphisms within *SFTPA *among additional racial and ethnic groups.

## Competing interests

The author(s) declare that they have no competing interests.

## Authors' contributions

MMP conceived of the study, analyzed and interpreted data, and drafted the manuscript. JFG participated in the analysis and interpretation of data, performed the statistical analysis, and helped revise the manuscript for important intellectual content. YZ was involved in the acquisition of data and provided technical support with the genotyping. EWT helped conceive the study, and was involved in the acquisition of data. KB conceived the study, participated in its design and coordination, and helped secure funding. TRH was involved in the critical revision of manuscript for important intellectual content and provided statistical expertise. MMB was involved in the study concept and design, study supervision, critical revisions of the manuscript for intellectual content, and helped obtain funding. BPL was involved in the study concept and design, study supervision, and obtained funding. All authors read and approved the final manuscript.

## Pre-publication history

The pre-publication history for this paper can be accessed here:



## References

[B1] Morgan WJ, Stern DA, Sherrill DL, Guerra S, Holberg CJ, Guilbert TW, Taussig LM, Wright AL, Martinez FD (2005). Outcome of asthma and wheezing in the first 6 years of life: follow-up through adolescence. American Journal of Respiratory & Critical Care Medicine.

[B2] Belanger K, Beckett W, Triche E, Bracken MB, Holford T, Ren P, McSharry JJE, Gold DR, Platts-Mills TA, Leaderer BP (2003). Symptoms of wheeze and persistent cough in the first year of life: associations with indoor allergens, air contaminants, and maternal history of asthma. American Journal of Epidemiology.

[B3] van Strien RT, Gent JF, Belanger K, Triche E, Bracken MB, Leaderer BP (2004). Exposure to NO2 and nitrous acid and respiratory symptoms in the first year of life. Epidemiology.

[B4] Ghildyal R, Hartley C, Varrasso A, Meanger J, Voelker DR, Anders EM, Mills J (1999). Surfactant protein A binds to the fusion glycoprotein of respiratory syncytial virus and neutralizes virion infectivity.. Journal of Infectious Diseases.

[B5] Benne CA, Kraaijeveld CA, van Strijp JA, Brouwer E, Harmsen M, Verhoef J, van Golde LM, van Iwaarden JF, Iino Y (1995). Interactions of surfactant protein A with influenza A viruses: binding and neutralization.. Journal of Infectious Diseases.

[B6] LeVine AM, Gwozdz J, Stark J, Bruno M, Whitsett J, Korfhagen T (1999). Surfactant protein-A enhances respiratory syncytial virus clearance in vivo. J Clin Invest.

[B7] LeVine AM, Kurak KE, Wright JR, Watford WT, Bruno MD, Ross GF, Whitsett JA, Korfhagen TR (1999). Surfactant protein-A binds group B streptococcus enhancing phagocytosis and clearance from lungs of surfactant protein-A-deficient mice. American Journal of Respiratory Cell & Molecular Biology.

[B8] Sano H, Kuroki Y (2005). The lung collectins, SP-A and SP-D, modulate pulmonary innate immunity. Molecular Immunology.

[B9] Hohlfeld JM (2002). The role of surfactant in asthma. Respiratory Research.

[B10] White RT, Damm D, Miller J, Spratt K, Schilling J, Hawgood S, Benson B, Cordell B (1985). Isolation and characterization of the human pulmonary surfactant apoprotein gene. Nature.

[B11] Fisher JH, Kao FT, Jones C, White RT, Benson BJ, Mason RJ (1987). The coding sequence for the 32,000-dalton pulmonary surfactant associated protein A is located on chromosome 10 and identifies two separate restriction-fragment length polymorphisms. American Journal of Human Genetics.

[B12] Kerr MH, Paton JY (1999). Surfactant protein levels in severe respiratory syncytial virus infection. American Journal of Respiratory & Critical Care Medicine.

[B13] Haataja R, Ramet M, Marttila R, Hallman M (2000). Surfactant proteins A and B as interactive genetic determinants of neonatal respiratory distress syndrome. Human Molecular Genetics.

[B14] Marttila R, Haataja R, Guttentag S, Hallman M (2003). Surfactant protein A and B genetic variants in respiratory distress syndrome in singletons and twins. American Journal of Respiratory and Critical Care Medicine.

[B15] Marttila R, Haataja R, Ramet M, Pokela ML, Tammela O, Hallman M (2003). Surfactant protein A gene locus and respiratory distress syndrome in Finnish premature twin pairs. Annals of Medicine.

[B16] Lofgren J, Ramet M, Renko M, Marttila R, Hallman M (2002). Association between surfactant protein A gene locus and severe respiratory syncytial virus infection in infants. Journal of Infectious Diseases.

[B17] Ramet M, Lofgren J, Alho OP, Hallman M (2001). Surfactant protein-A gene locus associated with recurrent otitis media. Journal of Pediatrics.

[B18] Pettigrew MM, Gent JF, Zhu Y, Triche EW, Belanger KD, Holford TR, Bracken MB, Leaderer BP (2006). Association of surfactant protein A polymorphisms with otitis media in infants at risk for asthma. BMC Medical Genetics.

[B19] Gent JF, Ren P, Belanger K, Triche E, Bracken MB, Holford TR, Leaderer BP (2002). Levels of household mold associated with respiratory symptoms in the first year of life in a cohort at risk for asthma.. Environmental Health Perspectives.

[B20] Pantelidis P, Lagan AL, Davies JC, Welsh KI, du Bois RM (2003). A single round PCR method for genotyping human surfactant protein (SP)-A1, SP-A2, and SP-D gene alleles. Tissue Antigens.

[B21] DiAngelo S, Lin Z, Wang G, Phillips S, Ramet M, Luo J, Floros J (1999). Novel, non-radioactive, simple and multiplex PCR-cRFLP methods for genotyping human SP-A and SP-D marker alleles. Disease Markers.

[B22] Saxton AM (2004). Genetic analysis of complext traits using SAS.

[B23] Carroll RJ, Ruppert D, Stefanski LA (1995). Measurement error in nonlinear models.

[B24] Harrod KS, Trapnell BC, Otake K, Korfhagen TR, Whitsett JA (1999). SP-A enhances viral clearance and inhibits inflammation after pulmonary adenoviral infection. American Journal of Physiology.

[B25] LeVine AM, Whitsett JA, Gwozdz JA, Richardson TR, Fisher JH, Burhans MS, Korfhagen TR (2000). Distinct effects of surfactant protein A or D deficiency during bacterial infection on the lung. Journal of Immunology.

[B26] Tino MJ, Wright JR (1996). Surfactant protein A stimulates phagocytosis of specific pulmonary pathogens by alveolar macrophages. American Journal of Physiology.

[B27] Allen MJ, Harbeck R, Smith B, Voelker DR, Mason RJ (1999). Binding of rat and human surfactant proteins A and D to Aspergillus fumigatus conidia.. Infection & Immunity.

[B28] Wang JY, Kishore U, Lim BL, Strong P, Reid KB (1996). Interaction of human lung surfactant proteins A and D with mite (Dermatophagoides pteronyssinus) allergens.. Clinical & Experimental Immunology.

[B29] Hohlfeld JM, Erpenbeck VJ, Krug N (2003). Surfactant proteins SP-A and SP-D as modulators of the allergic inflammation in asthma. Pathobiology.

[B30] Mishra A, Weaver TE, Beck DC, Rothenberg ME (2001). Interleukin-5-mediated allergic airway inflammation inhibits the human surfactant protein C promoter in transgenic mice. Journal of Biological Chemistry.

[B31] Wang JY, Shieh CC, You PF, Lei HY, Reid KB (1998). Inhibitory effect of pulmonary surfactant proteins A and D on allergen-induced lymphocyte proliferation and histamine release in children with asthma. American Journal of Respiratory & Critical Care Medicine.

[B32] Wang JY, Shieh CC, Yu CK, Lei HY (2001). Allergen-induced bronchial inflammation is associated with decreased levels of surfactant proteins A and D in a murine model of asthma. Clinical and Experimental Allergy.

[B33] Triche EW, Belanger K, Beckett W, Bracken MB, Holford T, Gent J, Jankun T, McSharry JJE, Leaderer BP (2002). Infant Respiratory Symptoms Associated with Indoor Heating Sources. American Journal of Respiratory and Critical Care Medicine.

[B34] Liu W, Bentley CM, Floros J (2003). Study of human SP-A, SP-B and SP-D loci: allele frequencies, linkage disequilibrium and heterozygosity in different races and ethnic groups.. BMC Genetics.

[B35] Eggleton P, Reid KB (1999). Lung surfactant proteins involved in innate immunity. Current Opinion in Immunology.

